# Normative Reference of the Single Leg, Medial Countermovement Jump in Adolescent Youth Ice Hockey Players

**DOI:** 10.3390/sports9080105

**Published:** 2021-07-26

**Authors:** Anthony S. Donskov, Jeffrey S. Brooks, James P. Dickey

**Affiliations:** 1 Department of Kinesiology, University of Western Ontario, London, ON N6A 357, Canada; jbrook45@uwo.ca (J.S.B.); jdickey@uwo.ca (J.P.D.); 2 Donskov Strength and Conditioning, Columbus, OH 43229, USA

**Keywords:** functional performance tests, ice skating, jumping, force plates

## Abstract

Functional performance tests provide quantitative information on specialized sport movements and are important for documenting training and fatigue. The single leg, medial countermovement jump provides objective measures of frontal plane force, velocity and power, and is relevant for ice hockey players given the similar lateral movement to ice skating. This study measured normative single leg, medial countermovement jump parameters (i.e., vertical and lateral maximum force, average concentric power and average concentric power during the last 100 ms) amongst male youth ice hockey players and assessed interlimb asymmetry in these healthy athletes. Ninety-one elite youth players participated in the study. Participants completed three right and three left jumps. Non-parametric tests were performed to evaluate between-jump and between-group comparisons. Many differences in jump force and power parameters were observed between the 10U/11U and 12U/13U age groups, and the 12U/13U and 14U/15U age groups, but differences were not as consistent between older or younger players. The average asymmetry index for each age group was less than 15% for force parameters, while the power parameters had larger asymmetry indices (between 9% and 22%). Our results provide age-specific reference values and asymmetry indices for male elite youth ice hockey players aged 10–18 years performing the single leg, medial countermovement jump.

## 1. Introduction

Ice hockey has become one of the most popular sports played in North America with 561,700 players under 18 years of age registered with USA Hockey in 2019–2020 [1]. As players mature, a greater emphasis is placed on their skill and physical development, resulting in improved upper body strength and lower body power [2]. Consequently, physical preparation training and testing is paramount for tracking progress and improvement over time [3]. Tests such as the countermovement jump, squat jump and three hop jump have been employed to measure physical performance [4,5]. However, the best tests for assessing physical capacities and return to sport are those that closely mimic the biomechanics of the sporting activity [6]. The single leg, medial countermovement jump is a reliable measure of assessing youth hockey player performance [7]. Nevertheless, normative values across multiple youth ice hockey age groups have yet to be reported.

The single leg, medial countermovement jump is a lower body power test that incorporates a high degree of force, velocity, impulsiveness and coordination in the frontal plane. It has been used to assess the unilateral power output of field and court sport athletes [8,9]. One study has examined the reliability of various temporal and kinetic variables involved in jumping vertically, horizontally and medially [8]. This study determined that eccentric and concentric peak force and concentric peak power were the only reliable measures between single leg vertical, horizontal and medial jumps [8]. Another group of researchers determined that single leg, countermovement jumping could differentiate between elite and non-elite soccer players [10]. Researchers reported that elite soccer players produced more peak vertical power than non-elite players during single leg jumps in the vertical, horizontal and medial directions, but these differences were not significant for bilateral jumps. They concluded that single leg jumping was more useful than the traditional bilateral countermovement jumping [10]. Single leg jumping is also appropriate for evaluating skating as it involves lateral propulsion on one leg. Velocity, force and power parameters describing performance of the single leg, medial countermovement jump demonstrate moderate to strong test re-test reliability in a group of U16 youth ice hockey players [7]. Accordingly, it is important to further explore the measurement properties and baseline normative values for the single leg, medial countermovement jump.

Interlimb asymmetry has also been explored for a variety of styles of single leg jumps in the vertical and horizontal directions [11,12]. Researchers determined that the single leg, vertical countermovement jump showed greater side-to-side differences compared with single, triple and crossover hops for distance, illustrating that single leg jumps may be particularly suited for assessing asymmetry. Asymmetry has important implications for performance as the degree of asymmetry in the single leg, countermovement jump was correlated with sprint times across distances of 5, 10 and 20 m in youth female soccer players [11]. Interlimb asymmetries are also inversely correlated with jumping ability [13]. Assessing differences in strength, power and performance between legs is important for skill and physical development as well as injury prevention and rehabilitation [14].

To the best of our knowledge, normative force and power values, as well as interlimb asymmetries of the single leg, medial countermovement jump, have not been reported for ice hockey players. These normative measures would be valuable for performance professionals, teachers and coaches to compare their athletes to normative baselines [15,16]. Furthermore, normative data about interlimb asymmetries may be useful for performance staff to monitor rehabilitation progress and return to play timelines [17,18]. Therefore, the purposes of this study were to measure normative single leg, medial countermovement jump parameters (i.e., maximum force, average concentric power and average concentric power during the last 100 ms) amongst youth ice hockey players, and to assess the interlimb asymmetry between legs in these healthy athletes. Our hypothesis was that all normative values would increase with age, similar to changes in jump performance with age [15,16].

## 2. Materials and Methods

### 2.1. Subjects and Study Design

A sample of convenience was used. In this study, 91 elite performing male youth ice hockey players from 10U, 11U, 12U, 13U, 14U, 15U, 16U and 18U teams participated. Group characteristics are provided in Table 1. All participants had medical clearance from a healthcare professional. Inclusion criteria for all subjects included no pre-existing medical conditions, no current lower or upper body musculoskeletal injuries, and currently participating in organized hockey. Testing for the 14U–18U age groups took place during the last month of the 2019–2020 hockey season, when training volume was low in preparation for league playoffs. Testing for the 10U–13U age groups took place at a training camp in August prior to the start of the 2020–2021 hockey season. Participants under the age of thirteen signed a written assent form, and their parents or guardians signed consent forms, prior to the study. Participants over the age of twelve gave written informed consent to participate in the study. The University of Western Ontario Health Science Research Ethics Board approved the experimental protocol (protocol 113858).

### 2.2. Procedures

Data collection was performed during windows of opportunity around restrictions due to COVID-19. This led to data collection occurring in two separate testing sessions, each at a different location. Testing for the 14U–18U players took place indoors at Donskov Strength and Conditioning (Columbus, OH, USA) training facility in February 2020. Testing for the 10U–13U players took place at the Ice Haus hockey rink (Columbus, OH, USA) in August 2020. A familiarization period was not provided prior to testing for the 14U–18U players; however, all participants were familiar with these jumps as they were part of their weekly in-season strength and conditioning plan. The 10U–13U players were familiarized with these jumps by their skill coaches as part of their weekly dynamic warm-ups. Each participant completed their testing in one session.

Participants performed a standardized 15 min warmup consisting of static stretching, mobility and dynamic movement (foam rolling, knee hugs, heel to butt, reverse lunge, single leg deadlift with reach, A skips, back pedaling, short accelerations). Participants performed single leg, medial countermovement jumps on both left and right legs for three repetitions each. Jumps were performed in randomized ordered blocks for each leg. Jump ground reaction forces were measured using bilateral force plates (OR6–7, AMTI, Watertown, MA, USA). A custom LabVIEW program (LabVIEW 2012, National Instruments, Austin, TX, USA) sampled the force plate signals at 200 Hz with a 16-bit analog-to-digital converter (USB 6211, National Instruments, Austin, TX, USA). One minute of rest was provided between each jump to prevent fatigue [19].

### 2.3. Jump Protocol

Standardized verbal commands and demonstrations were administered to all participants by the same staff member. Maximal effort on each jump attempt was encouraged by verbal support from the coaching staff. During the single leg, medial countermovement jump, participants squatted to a self-selected depth on the designated leg, while standing on a force plate, and then jumped medially as high and as far as possible landing on both legs. Arm swing was permitted. All jumps were monitored by two strength and conditioning professionals to ensure proper jumping technique. Compromised trials (improper technique, equipment malfunction) were discarded and repeated.

### 2.4. Data Processing

Force plate data was analyzed using custom software in LabVIEW. The force plate signals were unfiltered, similar to other jump research [20]. The resultant forces in the X, Y and Z directions were calculated as the sum of the X, Y and Z forces from both force plates. Participants’ bodyweight was extracted from a one second duration window from standing trials. Jump phases (initiation, transition between concentric and eccentric phases, end) were automated in LabVIEW and verified via visual inspection. Jump initiation was defined as the point of time where lateral force started to increase (10 N threshold). The point where the vertical force dropped to less than 10 N was defined as the end of the jump/task. The transition from the eccentric to the concentric phases was determined when the vertical velocity of the center of mass became positive for longer than 0.1 consecutive seconds, similar to other studies [8]. The product of velocity and force was used to calculate instantaneous vertical and lateral power, as performed in other studies [8,21]. Maximum force was extracted from the force-time curve. Average concentric power and average concentric power in the last 100 ms were extracted from the power curve. Force and power were expressed in raw units (N and W). In summary, the variables included vertical and lateral maximal force, average concentric power, and average concentric power during the last 100 ms. These variables are a subset of the parameters that were determined to be reliable in a recent study [7]. This subset was selected as these parameters assess independent constructs (based on low correlations). Jump performances were represented as the average of the parameters from the three jump trials.

### 2.5. Statistical Analysis

To account for outliers and uneven distributions within groups, non-parametric analyses were used [22]. Wilcoxon Sign tests compared left and right leg jump performance for all six variables within each of the four age groups. The false discovery rate method was used to control the familywise error rate for these tests, with a 0.05% threshold [23]. Data for the right and left sides was amalgamated if the differences between sides were not statistically significant. Kruskal–Wallis tests were used for between-group comparisons between age groups. Finally, Mann–Whitney U post hoc tests identified significantly different age pairs for each of the six jump parameters; only adjacent age groups (e.g., U10/U11 vs. U12/U13, U12/U13 vs. U14/U15) were compared and the false discovery rate method was used to control familywise error rate. Effect sizes and 95% confidence intervals were calculated for all adjacent age groups using the probability of superiority approach [24], and interpreted as values of 0.56, 0.64 and 0.71 corresponding to small, medium and large effect sizes [25]. Jump parameter characteristics are presented as box and whisker plots including the median, with boxes illustrating the first (Q1) and third quartiles (Q3), and whiskers are extended 1.5 times the length of the interquartile range beyond the box boundaries, defining the inner fence for identifying outliers [26].

Interlimb asymmetry index calculations were recorded for each participant and averaged for each team using the percentage difference between limbs calculation [27,28]. Statistical analysis was performed using Prism (V9.1.0, GraphPad Software LLC., San Diego, CA, USA).

## 3. Results

There was only one statistically significant difference (peak lateral force for the 12U/13U age group) between the right and left leg jump performances for any of the 24 comparisons (six force and power parameters for each of the four age groups) after false discovery rate adjustment. Consequently, all normative values were based on the combined right and left scores for each participant.

**Figure 1:**
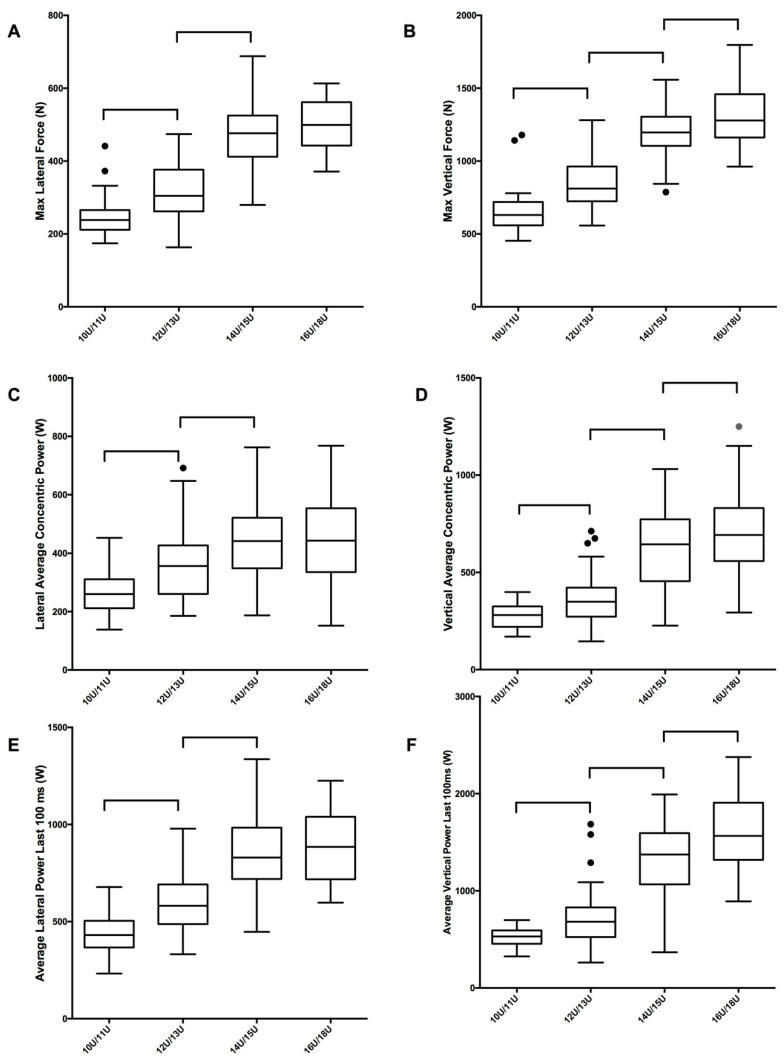
Box and whisker plots comparing youth ice hockey single leg, countermovement jump variables across each age group. Panels (**A**,**B**) illustrate the maximum lateral and vertical force, respectively. Panels (**C**,**D**) illustrate the lateral and vertical average concentric power, respectively. Panels (**E**,**F**) illustrate the average lateral and vertical power in the last 100 ms, respectively. Whiskers are extended 1.5 times the length of the interquartile range beyond the box boundaries, defining the inner fence for identifying outliers. Data points that are outside of these fences are identified with individual points. Significant differences (*p* < 0.05) between adjacent age groups are indicated with brackets. Note that the scales are different for the lateral and vertical panels.

In general, all of the power and force parameters for the single leg, medial countermovement jumps increased with age (Figure 1). Note that tabulated normative data are available as Supplementary Materials. The details of the statistical analysis and the effect sizes are reported in Table 2. Most parameters were significantly different between the ages of 10U/11U and 12U/13U, and 12U/13U and 14U/15U; the 14U/15U group outperformed the 12U/13U group by 22–44% in all six of the parameters. As a general trend, older (14U/15U and 16U/18U) age groups experienced fewer significant differences between age groups. The 12U/13U group had significantly larger scores than the 10U/11U group in all six performance parameters, outperforming them by 18–26%. In addition, three significant differences were observed between the 16U/18U age groups and the 14U/15U for maximum vertical force, vertical average concentric power, and average vertical power during the last 100 ms. The 16U/18U group outperformed the 14U/15U age group by 4–12% in five of the six performance parameters; however, only three were statistically significant (*p* < 0.05). The 14U/15U group outperformed the 16U/18U group by 1% for lateral average concentric power.

The average asymmetry index for each age group was less than 15% for both vertical and lateral force parameters (Table 3). The power parameters (vertical and lateral concentric power and the vertical and lateral average concentric power during the last 100 ms) had larger asymmetry indices than force parameters (maximum vertical and lateral force) for all age groups. These asymmetry indices varied between 9% and 22%. The maximum vertical and lateral force parameters had the lowest asymmetry magnitudes across all age groups. Asymmetry ranged from 3.7–10.4% for maximum vertical force. The 16U/18U group represented the lowest asymmetry index for both vertical and lateral force. Asymmetry values for maximum lateral force ranged from 3.7% for the 16U/18U group, to 4.5% for the 12U/U13 group.

## 4. Discussion

The purposes of this study were to measure normative single leg, medial countermovement jump parameters (i.e., maximum force, average concentric power and average concentric power during the last 100 ms) amongst youth ice hockey players, and to assess the interlimb asymmetry in these healthy athletes. These parameters were not significantly different between legs in our participants and therefore we defined our normative values based on the combined data set. We observed a general trend that most parameters increased between adjacent player age groups, partially supporting our hypothesis. In particular, we noted significant changes between the 10U/11U and 12U/13U, and 12U/13U and 14U/15U age groups, presumably related to physical maturation. Most asymmetries for these parameters were less than 15%.

The age-based normative data that we present in this paper provide a benchmark that performance professionals, teachers and coaches can use to evaluate their athletes. Our previous study reports a variety of measurement properties of the single-leg countermovement jump including standard error of measure, smallest real difference and typical error (and 90% confidence interval) [7]. We observed typical errors less than 5% for the vertical and lateral peak force parameter, less than 7.5% for the power in the last 100 ms parameter and less than 20% for the average concentric power parameter, which may help comparisons between athletes and these age-based normative data.

In general, it appears as though body mass plays a critical role in jump performance [15]. Body mass is associated with improved peak power in adolescent boys and girls [15]. The 16U/18U group was much heavier compared to the 10U/11U group. The 16U/18U age group outweighed the 10U/11U group by an average of 38.5 kg.

Access to a structured strength and conditioning plan also affects jump performance. This differentially affected the athletes in this study. Athletes in the 13U–18U age groups participated in a structured strength and conditioning plan during the hockey season. Structured weight training has been shown to increase countermovement performance due to an increase in cross sectional area and muscle mass which leads to larger force output [29,30]. Accordingly, the fact that the younger age groups (10U–12U) did not partake in regular strength and conditioning sessions may have affected jump performance.

A large study reported increases in countermovement jump height between 10–11 year old, 12–14 year old and 15–17 year old males [31]. However, our data have greater granularity as we evaluated differences between four age groups (except for the 17U age group that included 17- and 18-year-olds). We observed the greatest number of differences between the 10U/11U and 12U/13U, and the 12U/13U and 14U/15U age groups. These age groups are approaching peak height growth velocity for boys (13.85 ± 0.65 years old) [32]. During this age range tasks such as speed, static strength and power are related to the ages that an athlete matures [33]. Specifically, the window of time between the ages of 12U/13U and 14U/15U showed the largest change in jump performance in our group of youth ice hockey players as six of six performance parameters had significant differences and as much as a 22–44% increase in performance compared with the 12U/13U age group.

Finally, the asymmetry indices were lower for the vertical and lateral force parameters compared to the power parameters. The average vertical asymmetry magnitudes for vertical force were 4.27% for all age groups, while the overall lateral force asymmetry was 7.37%. These asymmetries are consistent with previous research on elite youth soccer players [11]; however, that study calculated asymmetry of the jump height parameter measured with the “My Jump” iPhone application, and therefore may not be directly comparable. The soccer paper reports that asymmetry has important implications for performance as the degree of asymmetry in the single leg, countermovement jump was correlated with sprint times across various distances [11]. Vertical and lateral power asymmetries in the current paper were larger during the single leg, medial countermovement jump as magnitudes varied between 9.11% to 22.60%. These values are difficult to compare as normative power parameters for youth ice hockey players do not currently exist to the authors’ knowledge. Previous research on competitive male soccer players has evaluated asymmetry during countermovement jumps [17]. They suggest that asymmetries larger than 15% may be considered abnormal. However, it is important to note that they measured peak vertical force during countermovement jumps, and accordingly this threshold may not be relevant for the lateral force, vertical power and lateral power parameters that we report in this study.

### Limitations

There are limitations to this study. This study tested youth athletes playing in a single youth hockey organization. Different organizations may have different resources such as strength and conditioning programming which may affect jump performance. We included players of different playing positions which may have affected our results. Further research should evaluate whether there are differences in single leg, medial countermovement jump performance between player positions. A sample of convenience was used, however, we observed statistically significant differences between adjacent age groups for many of our parameters, and large effect sizes between adjacent age groups for many of our parameters, which indicates that this experiment had adequate statistical power.

## 5. Conclusions

We determined normative values for parameters from the single leg, medial countermovement jump for male youth hockey players 10U–18U. This is an important performance test for monitoring strength and conditioning in hockey players. The normative data presented in this paper serves as a baseline for evaluating jump performance in youth hockey players. The single leg, medial countermovement jump allows the tester to measure ground reaction forces and power in the frontal plane which makes this test a relevant tool in all phases of sport performance and rehabilitation. To our knowledge, this is the first study to generate normative values of these jump parameters, and the first to investigate interlimb asymmetries in youth ice hockey players.

## Figures and Tables

**Table 1:** Demographics (mean ± SD) for the youth ice hockey age groups.

Group	Number *	Age (Years)	Height (cm)	Mass (kg)
10U/11U	21	10.4 ± 0.4	145.2 ± 5.9	37.7 ± 5.9
12U/13U	25	12.3 ± 0.4	158.2 ± 6.8	47.5 ± 8.3
14U/15U	26	14.6 ± 0.5	174.7 ± 5.3	70.4 ± 8.9
16U/18U	19	16.8 ± 0.5	180.4 ± 6.8	76.2 ± 9.6

* Number of participants in each ice hockey age group.

**Table 2:** Median difference, statistical significance and effect sizes of adjacent age group comparisons.

Age Groups	Hodges–Lehmann Median Difference	m	n	U	False Discovery Rate Threshold	*p*-Value *	Effect Size (95% CI)
VERT Avg Con Power (W)							
10U/11U vs. 12U/13U	70.55	31	49	426	0.033	0.0008 ❖	0.720 ►►► (0.592–0.816)
12U/13U vs. 14U/15U	267.3	49	52	306	0.017	<0.0001 ❖	0.880 ►►► (0.792–0.932)
14 U/15U vs. 16U/18U	88.85	52	38	744	0.05	0.046 ❖	0.624 ► (0.502–0.729)
VERT Avg Con Power 100 ms (W)							
10U/11U vs. 12U/13U	160.2	31	49	383	0.033	0.0001 ❖	0.748 ►►► (0.622–0.839)
12U/13U vs. 14U/15U	647.4	49	52	264	0.017	<0.0001 ❖	0.896 ►►► (0.812–0.944)
14 U/15U vs. 16U/18U	268.8	52	38	613	0.05	0.002 ❖	0.690 ►► (0.570–0.786)
LAT Avg Con Power (W)							
10U/11U vs. 12U/13U	80.62	31	49	397	0.017	0.0003 ❖	0.739 ►►► (0.612–0.832)
12U/13U vs. 14U/15U	93.22	49	52	748	0.033	0.0003 ❖	0.706 ►► (0.596–0.794)
14 U/15U vs. 16U/18U	2.10	52	38	981	0.05	0.958	0.504 (0. 386–0.621)
LAT Avg Con Power 100 ms (W)							
10U/11U vs. 12U/13U	144.0	31	49	293	0.017	<0.0001 ❖	0.807 ►►► (0.688–0.885)
12U/13U vs. 14U/15U	253.8	49	52	356	0.033	<0.0001 ❖	0.860 ►►► (0.768–0.917)
14 U/15U vs. 16U/18U	43.23	52	38	875	0.05	0.360	0.557 ►►► (0.437–0.670)
MAX VERT Force (N)							
10U/11U vs. 12U/13U	181.2	31	49	293	0.017	<0.0001 ❖	0.807 ►►► (0.688–0.885)
12U/13U vs. 14U/15U	371.7	49	52	190	0.033	<0.0001 ❖	0.657 ►► (0.544–0.752)
14 U/15U vs. 16U/18U	89.83	52	38	698	0.05	0.0175 ❖	0.647 ►► (0.526–0.749)
MAX LAT Force (N)							
10U/11U vs. 12U/13U	64.85	31	49	310	0.017	<0.0001 ❖	0.796 ►►► (0.675–0.876)
12U/13U vs. 14U/15U	157.8	49	52	202	0.033	<0.0001 ❖	0.921 ►►► (0.843–0.960)
14 U/15U vs. 16U/18U	33.60	52	38	756	0.05	0.0583	0.617 ►►► (0.496–0.724)

m, n and U refer to the number of participants in the two groups and the value of the Mann–Whitney U statistic. VERT refers to vertical, LAT refers to lateral, Con refers to concentric. * *p* value for the Mann–Whitney U test, before considering the false discovery rate adjustment ❖ denotes statistically significant differences at *p* < 0.05 after the false discovery rate adjustment. ► denotes a small effect size, ►► denotes a medium effect size, ►►► denotes a large effect size.

**Table 3:** Mean (SD) Percent Asymmetry Index (%) for each jump parameter, per each individual age group.

Age Group	VERT Avg Con Power	VERT Avg Con Power (100 ms)	LAT Avg Con Power	LAT Avg Con Power (100 ms)	Max VERT Force	Max LAT Force
10U/11U	13.89 (7.50)	16.67 (8.20)	12.75 (7.25)	14.44 (5.46)	4.32 (1.97)	7.12 (3.33)
12U/13U	12.89 (8.86)	15.00 (10.27)	18.77 (12.76)	14.07 (11.50)	4.57 (3.50)	10.45 (9.56)
14U/15U	21.53 (11.94)	19.22 (11.89)	22.60 (15.52)	10.32 (8.04)	4.46 (3.30)	5.82 (4.31)
16U/18U	14.48 (10.85)	12.33 (5.83)	22.17 (16.15)	9.11 (7.33)	3.74 (2.78)	6.05 (4.82)

VERT refers to vertical, LAT refers to lateral, Con refers to concentric.

## Data Availability

The data presented in this study are available on request from the corresponding author.

## Supplementary Materials

The following are available online at https://www.mdpi.com/article/10.3390/sports9080105/s1, File S1. Tabulated normative data for each age group.
